# Hypoxic Burden in Children With Sleep‐Disordered Breathing: Determinants and Correlates

**DOI:** 10.1111/jsr.70211

**Published:** 2025-09-20

**Authors:** Plamen Bokov, Benjamin Dudoignon, Christophe Delclaux

**Affiliations:** ^1^ Université de Paris‐Cité, AP‐HP, Hôpital Robert Debré Service de Physiologie Pédiatrique‐Centre du Sommeil, INSERM NeuroDiderot Paris France

**Keywords:** apnea, intermittent hypoxia, paediatric, sleepiness, snoring

## Abstract

Hypoxic burden (HB) is an emerging metric for quantifying intermittent hypoxia associated with sleep apnea, offering potential advantages over traditional measures such as the apnea‐hypopnea index (AHI). This study evaluated the distribution and clinical significance of non‐respiratory event‐specific HB in children and adolescents with habitual snoring, exploring its relationship with sleepiness and other clinical parameters. The data were gathered from 512 children referred for suspected sleep‐disordered breathing (SDB), focusing on 380 subjects with available HB data. HB was calculated as the total area under oxygen saturation (SpO_2_) curves for events with ≥ 3% oxygen desaturation, with a median value of 1.7% min/h [IQR: 0.6% min/h–4.6% min/h]. Children with moderate‐to‐severe obstructive sleep apnea syndrome (OSAS) exhibited significantly higher HB than those with mild OSAS or primary snoring (7.5% min/h, 2.7% min/h, and 1.1% min/h, respectively). HB was notably linked to the AHI (rho_s_ = 0.61), the oxygen desaturation index (rho_s_ = 0.73), and minimum SpO_2_ (rho_s_ = −0.70). Furthermore, increased HB was observed in obese children and those with tonsillar hypertrophy, underscoring their synergistic impact. These effects were reflected in desaturation depth rather than their duration. In children with primary snoring, HB showed a significant association with parent‐reported sleepiness. Specifically, there was a 39% increase in the odds ratio for a modified Epworth sleepiness scale score exceeding 10 for each standard deviation increase in HB (*p* = 0.040). These findings suggest HB as an independent marker of SDB severity, with potential implications for understanding cognitive deficits linked to SDB in children.

**Abbreviations**
AHI: apnea‐hypopnea index
BMI: body‐mass index
HB: hypoxic burden
mESS: modified Epworth Sleepiness Scale
(N)REM: (non‐) rapid eye movement
OAHI: obstructive apnea‐hypopnea index
ODI_3%_
: 3% oxygen desaturation index
OSAS: obstructive sleep apnea syndrome
PSG: polysomnography
SD: standard deviation
SDB: sleep disordered breathing
SpO_2_
: peripheral oxygen saturation
T90: total sleep time with oxygen saturation < 90%

## 1 Introduction

Hypoxic burden (HB) is a novel metric for assessing intermittent hypoxia associated with obstructive sleep apnoea syndrome (OSAS). HB is defined as the total area under the peripheral oxygen saturation (SpO_2_) curve from a pre‐event baseline. Various measures and definitions of HB have been proposed and studied (Parekh 2024). They demonstrate potential in overcoming the limitations of the apnea‐hypopnea index (AHI) and offer greater prognostic value (Martinez‐Garcia et al. 2023). In adults, HB has been identified as an independent predictor of cardiovascular morbidity. Azarbarzin et al. (2019), using two large community‐based cohort studies, observed that, unlike the AHI, the HB strongly predicted cardiovascular mortality. HB was also shown in a community‐based dataset to be linked to hypertension risk (Kim et al. 2020). These authors found that for every one standard deviation (SD) increase in log‐transformed HB, there was a 0.9% increase in diastolic blood pressure (95% CI: 0.3–1.6, *p* = 0.004). However, there was no association with systolic blood pressure. Recently, we also found that higher levels of HB were associated with higher blood pressure levels in childhood OSAS, independent of the AHI (Bokov, Dudoignon, and Delclaux 2025).

Several methods and definitions have been proposed to calculate HB, which can be broadly categorised into respiratory event‐specific and non‐respiratory event‐specific measures (Parekh 2024). The approach developed by Azarbarzin and colleagues utilises manually marked respiratory events as a precursor for identifying candidate desaturation events. These events are then analysed using an area‐based calculation, termed HB, measured in percent (%) times minutes per hour of sleep (Azarbarzin et al. 2019). In contrast, other researchers have proposed a non‐respiratory event‐specific HB measure (Parekh et al. 2023) that considers both the left and right peaks of a candidate desaturation event and calculates the area bounded by the SpO_2_ nadir. This fully automated method accounts for disconnects or noisy signals and has also been shown to be a stronger predictor of cardiovascular mortality than the AHI (Parekh et al. 2023).

Overall, it is thought that hypoxia‐dominant OSAS, a subtype of OSA in which ventilatory changes during the night result in oxygen desaturation and increased HB (whatever the method of calculation), but not necessarily arousal from sleep, can increase vascular inflammation, sympathetic nervous system activity, and as a result lead to an increased risk for cardiovascular diseases.

Intermittent hypoxia has also been shown to be associated with oxidative injury, which in turn is linked to neuronal damage and degeneration in wake‐promoting regions of the brain (Lal et al. 2021). A positive correlation between HB and the Epworth sleepiness scale (ESS) score has been reported in adults (Esmaeili et al. 2023; Kainulainen et al. 2019). Thus, the determinants and correlates, such as sleepiness, of HB deserve to be studied, especially in children in whom limited data are available on HB.

This study assessed the significance and distribution of non‐respiratory event‐specific HB in children and adolescents with suspected sleep disordered breathing (SDB). Additionally, it explored the relationships between HB and the clinical characteristics of OSAS in children, as well as its connection to morbidity related to SDB, particularly excessive daytime sleepiness.

## 2 Methods

### 2.1 Participants and Ethical Issues

We included children aged 3–18 years with habitual snoring, defined as snoring occurring most of the night on at least three nights per week for a minimum of 3 months, who were referred to our facility for suspected OSAS. The data collected included demographic information, ethnicity, body mass index (BMI) *z*‐score (calculated using height and weight measured by a nurse), and symptoms related to snoring and/or SDB. Some children had their polysomnography (PSG) performed at another institution and were referred to us for treatment initiation. Non‐inclusion criteria for the study were the presence of a syndromic disease, neurological disability, current medical treatment, or any disease affecting oxygen levels in the blood, except for asthma.

On the same day as their evaluation, each child underwent a clinical examination followed by an overnight polysomnography. Tonsil size was assessed using the Brodsky scale (Brodsky 1989), tonsillar hypertrophy was defined as a Brodsky grade of 3 or 4, and excessive daytime sleepiness was evaluated using the modified ESS (Snow et al. 2002).

Obesity was defined as a BMI *z*‐score > 3 for children aged < 5 years or a BMI *z*‐score > 2 for children 5 years and older (de Onis et al. 2007).

This study was approved by the local ethics committee (PHENOSAS: No. 2018‐416). The database of the collected data was registered with the French regulatory agency (CNIL). The participants and their parents were informed of the collection of the prospective data for research purposes, and they were given the option to opt out of the study in compliance with French law governing non‐interventional observational research. The study complied with the STROBE guidelines for cross‐sectional studies.

### 2.2 In‐Laboratory Polysomnography

Overnight polysomnography studies were conducted either in‐laboratory for children under 8 years old or via ambulatory setups for children aged 8 years and older. These studies utilised an Alice 6 LDx or PDX polysomnography system (Philips, Murrysville, PA, USA) or a Somté PSG system (Compumedics, Australia), as previously described (Bokov et al. 2023). The AHI was calculated using all apnea and hypopnea events associated with desaturations ≥ 3% or arousal. SpO_2_ signals were captured by fingertip pulse oximetry (Nonin, Plymouth, Minnesota) and digitally sampled at 1 Hz. For in‐laboratory studies, the recordings were supplemented with infrared video monitoring. The sleep data were scored according to standard paediatric sleep scoring criteria by experienced paediatric sleep physicians (Berry et al. 2017). Eighty‐nine children were referred to our clinic with a prior diagnosis of moderate to severe OSAS, and their sleep study data were unavailable. We used the following definitions for SDB. Primary snoring was considered as obstructive AHI (OAHI) of less than 2 episodes/h of sleep; mild OSAS as OAHI of 2–5 episodes/h of sleep; and moderate‐to‐severe OSAS as OAHI exceeding five episodes/h of sleep (Kaditis et al. 2016). The oxygen desaturation index was defined as the number of times per hour that blood oxygen saturation decreased by at least 3% (ODI_3%_) from baseline.

### 2.3 Non‐Respiratory Event‐Specific Hypoxic Burden

HB was defined as the total area under the desaturation curve associated with ≥ 3% desaturation events, which were automatically identified. Prior to calculating the total area under the desaturation curve, the SpO_2_ signal was pre‐processed as follows. Periods of wake identified using manually scored sleep/wake states were discarded. Artefacts indicating non‐physiological values, such as consecutive decreases in SpO_2_ exceeding 5%, were also removed. An event was identified by the progressive decrease in SpO_2_ of at least 2% over 6 s or less. SpO_2_ nadirs were then identified, and the depth of desaturation was determined by subtracting the nadir SpO_2_ from the baseline saturation prior to the event. The sensitivity of our algorithm to noisy signals has been tested, and the results are presented in Supporting Information S1.

Unlike the approach by (Azarbarzin et al. 2019), we did not use the pre‐event baseline saturation defined as the maximum SpO_2_ during the 100 s before the event's end. This decision avoided falsely elevated baselines resulting from overshoots caused by overcorrection of SpO_2_ following a previous desaturation. The HB was computed by summing the individual desaturation areas and normalising the total area by the sleep duration, with HB expressed in units of % min/h. The HB was calculated for the entire sleep period.

### 2.4 Modified Epworth Sleepiness Scale

The modified Epworth sleepiness scale (mESS) score was derived from one of the most common self‐reported instruments for the assessment of daytime sleepiness, the ESS. The scale was modified for children by including more age‐appropriate behaviours, such as those while being at school; parents were asked to provide responses to these questions. The item “falling asleep while driving a car” was changed to “falling asleep at school” (Snow et al. 2002). Each hypothetical situation was scored on a scale of increasing likelihood of nodding off or falling asleep: (0) none, (1) slight, (2) moderate, and (3) high. The scores from each of the eight items were summed, resulting in a total score ranging from 0 to 24, with a score above 10 considered excessively sleepy (Melendres et al. 2004).

### 2.5 Statistical Analyses

The normality of the data was assessed using the Shapiro–Wilk test. The results are reported as medians [25th; 75th percentiles]. Continuous variables were compared between groups using either the *t*‐test or the Wilcoxon test when the assumption of normality was violated, or using one‐way ANOVAs or the Kruskal‐Wallis test when more than two groups were compared. Categorical variables were analysed using the Chi‐square or Fisher's exact test, as appropriate. Correlations were examined using Spearman's rank correlation coefficient (rho). A Box‐Cox transformation was applied for non‐normally distributed variables included in the multivariate analysis. This transformation followed the form:

$$y^{\left(\lambda \right)}=\left(y^{\lambda }-1\right)/\lambda$$

The *λ* values that facilitated the normalisation of distributions were as follows: −1 for desaturation duration, −0.5 for HB and AHI, and 0.5 for desaturation depth and age. The desaturation depth was calculated using the approximated formula for subjects with moderate to severe OSAS: 2*HB/(AHI*desaturation duration) (Azarbarzin et al. 2019).

Subsequently, we used a linear mixed‐effect model to examine the individual contributions of HB to the mESS score, with the “subject” included as a random effect. We conducted these analyses on the entire group of subjects, as well as individually on the primary snoring children, using as independent predictors of mESS variables that have been associated with sleepiness in children (age (Janssen et al. 2017), ethnicity (Philbrook et al. 2018), BMI (Gozal et al. 2001), asthma (van Maanen et al. 2013), female sex (Janssen et al. 2017; Selvadurai et al. 2022), AHI (Bacon et al. 2024; Melendres et al. 2004), and periodic limb movement index (PLMI) (Melendres et al. 2004)). In the fully adjusted model, covariates included age, ethnicity, asthma status, obesity, PLMI, and sex. Additional statistical analyses are described in the text. All *p* values reported are two‐tailed, with statistical significance set at < 0.05. All statistical analyses were performed with R software version 4.4.1.

## 3 Results

HB data were available for 380 out of the 512 children. For the remaining 132 children, the quality of the oximetry recordings was insufficient for HB calculation, or the oximetry data were unavailable due to polysomnography being performed at another institution. There were no significant differences between the two groups of children (see Table S1), except for a more severe desaturation index in the group with unexploitable oximetry. This can be explained by the fact that 89 children in that group, who had an overrepresentation of severe OSAS, had their polysomnography conducted at another institution. In the remaining population, the median HB was 1.7% min/h [25th–75th percentile: 0.6% min/h–4.6% min/h], with extreme values ranging from 0% min/h to 156.7% min/h. In Table 1, we present the distribution of patients and their clinical characteristics according to the severity of SDB.

**TABLE 1:** Cohort demographic and sleep characteristics (*N* = 380) of the children according to the severity of the sleep‐disordered breathing.

Characteristic	Primary snoring *n* = 225	Mild OSAS *n* = 56	Moderate‐to‐severe OSAS *n* = 99	*p*
Age, years	10.0 [7.3; 12.8]	11.6 [8.7; 14.6]	11.7 [8.7; 14.1]	0.003
Sex (male %)	119 (53%)	32 (57%)	58 (59%)	0.600
Ancestry, C/Af/A/M	158/51/2/14	23/28/3/2	43/37/8/11	< 0.001
BMI *Z*‐score	1.60 [0.39; 2.36]	2.30 [1.41; 2.60]	2.28 [1.16; 2.66]	< 0.001
Obese, *n* (%)	91 (40%)	35 (63%)	59 (60%)	< 0.001
Asthma, *n* (%)	57 (25%)	14 (25%)	18 (18%)	0.396
Allergy, *n* (%)	71 (32%)	23 (41%)	24 (24%)	0.109
HB, min%/h	1.1 [0.4; 2.2]	2.7 [0.8; 5.1]	7.5 [3.0; 15.9]	< 0.001
AHI, h^−1^	1.1 [0.6; 1.6]	3.5 [3.1; 4.7]	12.4 [8.5; 20.9]	< 0.001
OAHI, h^−1^	0.4 [0.2; 0.9]	3.0 [2.5; 3.5]	11.4 [7.5; 19.8]	NT
ODI_3%_, h^−1^	0.9 [0.3; 1.7]	3.0 [1.7; 4.3]	8.4 [4.6; 17.3]	< 0.001
SpO_2_ min, %	93 [91; 94]	90 [89; 92]	87 [83; 90]	< 0.001
T90, %	0.0 [0.0; 0.0]	0.0 [0.0; 0.0]	0.0 [0.0; 0.3]	< 0.001
Arousal index, h^−1^	6.8 [4.8; 8.9]	8.2 [5.3; 10.5]	14.0 [9.2; 19.8]	< 0.001
Sleep quality				
NREM 1, % TST	5.6 [3.2; 8.4]	5.6 [4.0; 7.8]	7.1 [3.6; 12.0]	0.026
NREM 2, % TST	44.6 [37.5; 51.9]	44.4 [35.2; 51.3]	41.6 [33.9; 49.8]	0.527
NREM 3, % TST	27.8 [23.1; 35.0]	30.9 [23.5; 36.3]	28.3 [23.0; 35.9]	0.689
REM, % TST	20.5 [17.7; 23.6]	19.6 [17.6; 24.2]	19.3 [16.7; 22.8]	0.073
Brodsky grade, 0/1/2/3/4^(missing data)^	70/81/48/21/3^(2)^	13/17/15/7/4	16/27/15/29/12	< 0.001
Sleep questionnaires				
mESS	8 [4; 11]	7 [4; 10]	6 [3; 10]	0.466

*Note*: Ethnicities are Caucasian/African‐Caribbean/Asian/Mixed.

Abbreviations: (N)REM, (non‐) rapid eye movement; AHI, apnea–hypopnea index; BMI, body mass index; HB, hypoxic burden; mESS, modified Epworth Sleepiness Scale; NT, not tested; OAHI, obstructive apnea‐hypopnea index; ODI_3%_, 3% oxygen desaturation index; SpO_2_, peripheral oxygen saturation; T90, total sleep time with oxygen saturation < 90%.

All the respiratory parameters, including HB, AHI, OAHI, oxygen desaturation index (ODI_3%_), SpO_2_ minimum, and time spent under 90% of saturation (T90), showed significant differences between the three groups.

Correlations between HB and conventional polysomnography variables were calculated. Highly significant (*p* < 0.001) correlations were observed with the AHI (rho_s_ = 0.61), OAHI (rho_s_ = 0.55), ODI_3%_ (rho_s_ = 0.73), SpO_2_ minimum (rho_s_ = −0.70), and T90 (rho_s_ = 0.50). Similar correlations were observed when analysing only children with moderate‐to‐severe OSAS, with rho_s_ = 0.58, rho_s_ = 0.58, rho_s_ = 0.83, rho_s_ = −0.70, and rho_s_ = 0.62, respectively.

**FIGURE 1:**
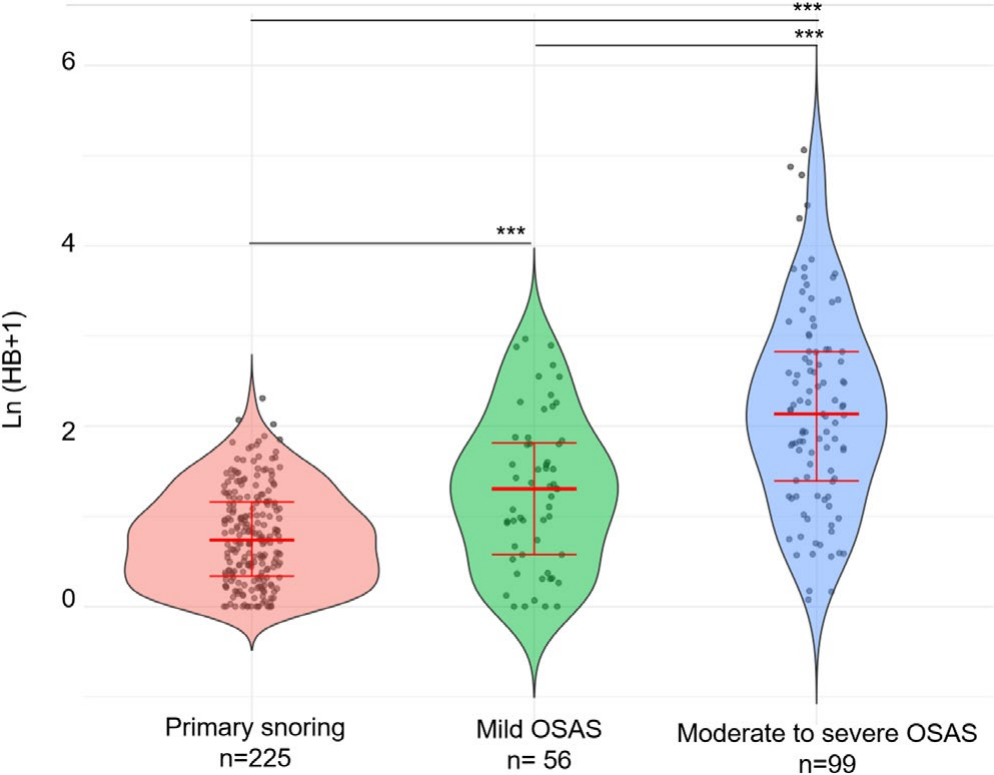
Hypoxic burden according to the severity of sleep disordered breathing. Primary snoring defined as an OAHI < 2/h, mild OSAS (2/h ≤ OAHI ≤ 5/h), and moderate‐to‐severe OSAS (OAHI > 5/h). Thick red bars—medians, vertical red error bars—25th–75th percentile. The group comparisons and their significance are presented by the horizontal lines and the asterisks. ***p < 0.001. OAHI, obstructive apnea‐hypopnea index; OSAS, obstructive sleep apnea syndrome.

**FIGURE 2:**
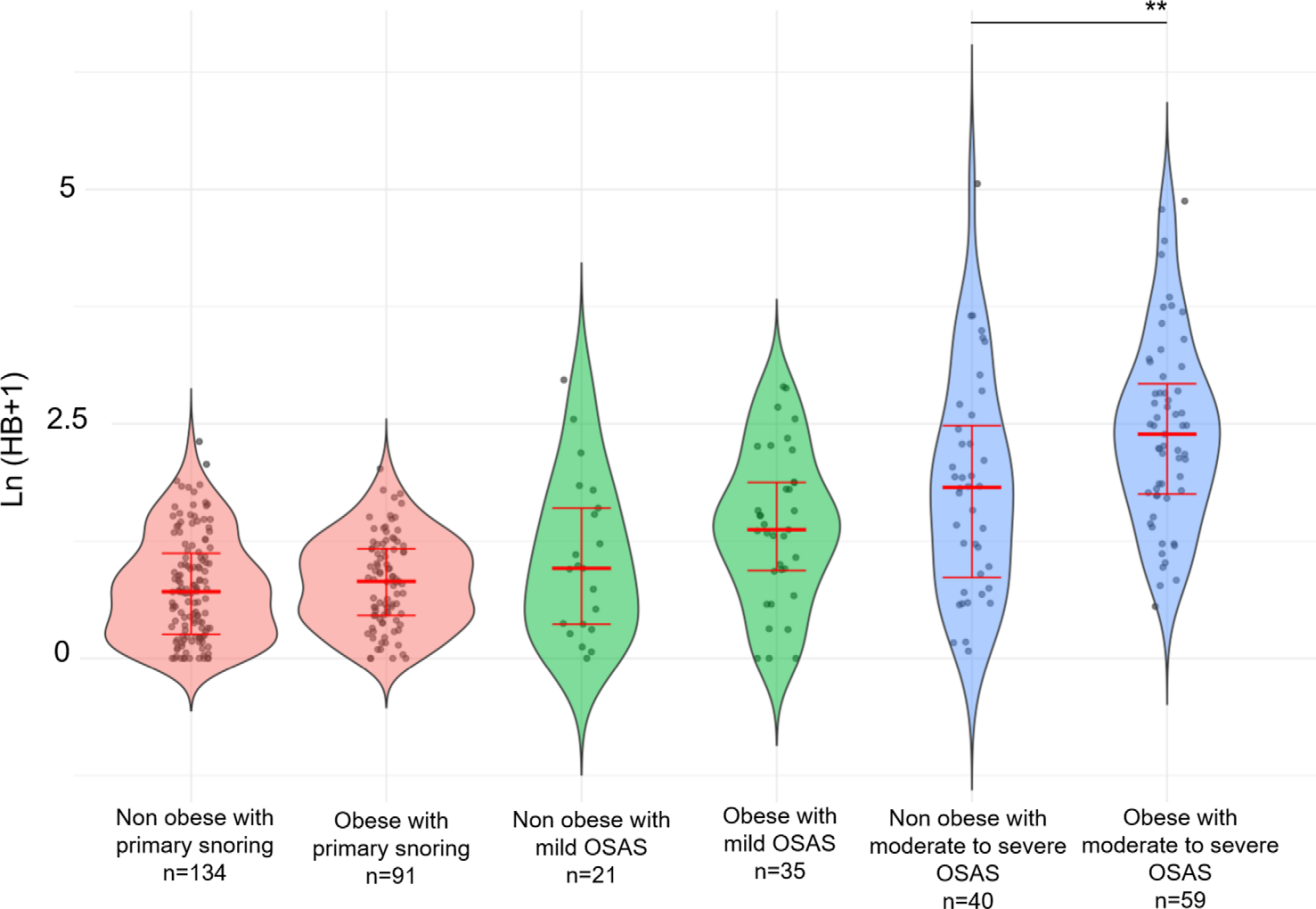
Hypoxic burden according to the severity of sleep disordered‐breathing and obesity. Primary snoring defined as an OAHI < 2/h, mild OSAS (2/h ≤ OAHI ≤ 5/h), and moderate‐to‐severe OSAS (OAHI > 5/h). Thick red bars—medians, vertical red error bars—25th–75th percentile. The only significant difference between the groups of the same level of SDB severity is represented by a horizontal line and the asterisks. **p < 0.01. OAHI, obstructive apnea‐hypopnea index; OSAS, obstructive sleep apnea syndrome.

**FIGURE 3:**
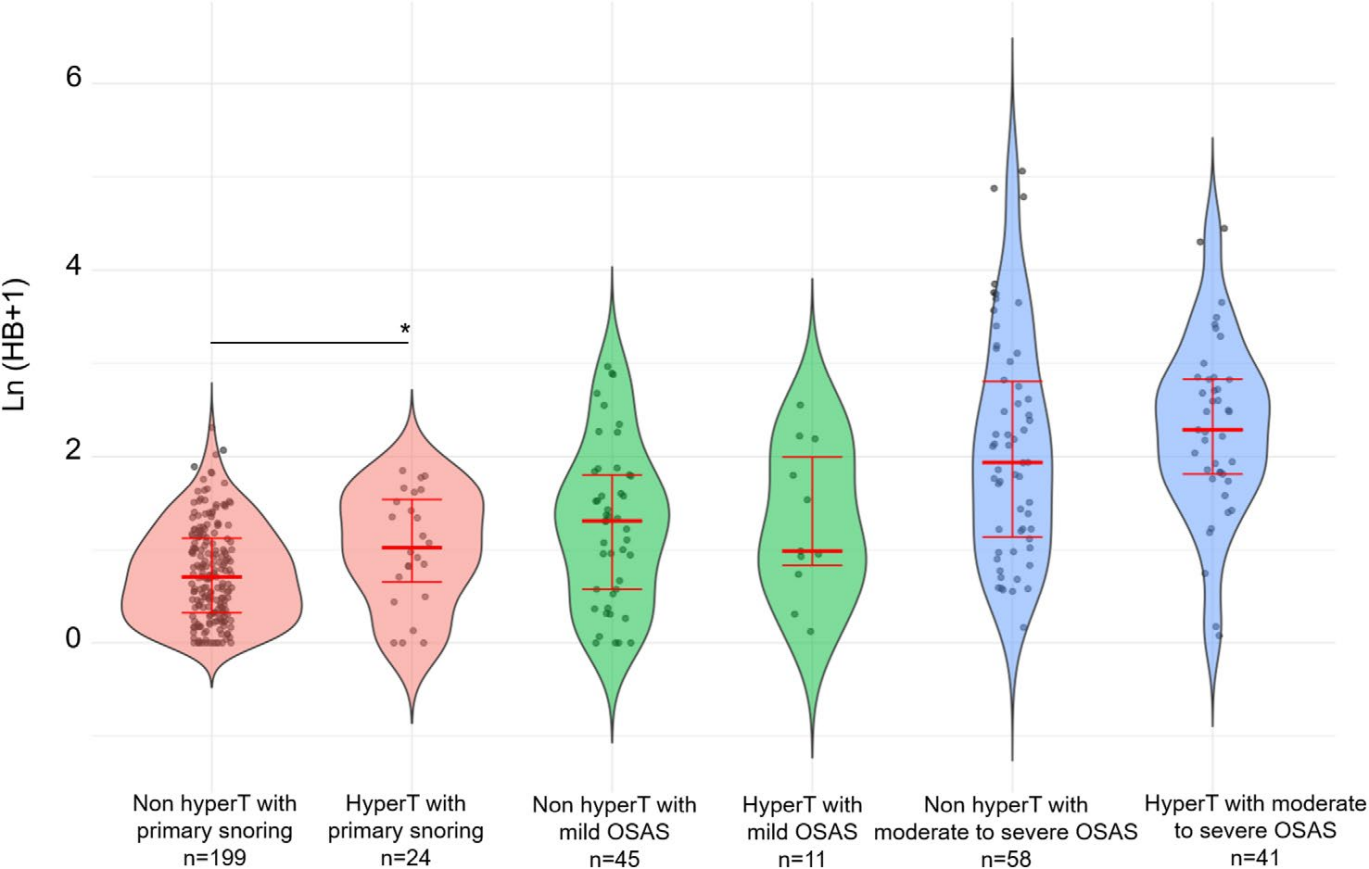
Hypoxic burden according to severity of sleep disordered breathing and tonsil hypertrophy. HyperT is tonsil hypertrophy (Brodsky grade > 2). Primary snoring defined as an OAHI < 2/h, mild OSAS (2/h ≤ OAHI ≤ 5/h), and moderate‐to‐severe OSAS (OAHI > 5/h). Thick red bars—medians, vertical red error bars—25th–75th percentile. The only significant difference between the groups of the same level of SDB severity is represented by a horizontal line and the asterisk. The Brodsky grade was not available for two patients from the primary snoring group. *p < 0.05. OAHI, obstructive apnea‐hypopnea index; OSAS, obstructive sleep apnea syndrome.

The distribution of HB based on the severity of OSAS or primary snoring is illustrated in (Figure 1 and Table 1). The combined impact of obesity and SDB on the HB is shown in Figure 2. The effect of tonsillar hypertrophy and SDB is depicted in Figure 3. Notably, the additive effect of obesity on the HB was most prominent in children with moderate‐to‐severe OSAS, whereas the impact of tonsillar hypertrophy was most significant in children with primary snoring. In detail, the HB of children with primary snoring according to the presence of obesity was 1.04 [0.3; 2.07] in non‐obese versus 1.27 [0.58; 2.22] % min/h in obese children (*p* = 0.263). In children with mild OSAS, the HB was 1.61 [0.44; 3.96] in non‐obese versus 2.94 [1.55; 5.52] % min/h in obese children (*p* = 0.141), while in children with moderate to severe OSAS the respective values were 5.19 [1.37; 10.97] versus 9.89 [4.76; 17.70] % min/h (*p* = 0.007).

Similarly, the results were 1.03 [0.38; 2.08] versus 1.79 [0.93; 3.66] % min/h (*p* = 0.031) in children with primary snoring, 2.70 [0.78; 5.07] versus 1.68 [1.31; 6.49] % min/h (*p* = 0.992) in children with mild OSAS, and 5.93 [2.12; 15.51] versus 8.84 [5.13; 15.90] % min/h (*p* = 0.153) in children with moderate to severe OSAS, according to the absence or the presence of hypertrophied tonsils, respectively.

Supplementary analysis revealed that both obesity and tonsillar hypertrophy were independent determinants of the HB (Table 2) with comparable contributions. Further investigations into whether obesity and tonsillar hypertrophy are linked to desaturation duration or desaturation depth in children with moderate‐to‐severe OSAS showed that the desaturation depth was independently increased in the obese patients (*p* = 0.013), but not in children with tonsillar hypertrophy (*p* = 0.153). Desaturation duration was not significantly influenced by obesity (*p* = 0.608) or tonsillar hypertrophy (*p* = 0.990) (see Table S2).

**TABLE 2:** Explanatory models for HB.

Adjusted‐*R* ^2^	Covariate coefficients (standardised beta), 95% CI	*p*
Model 1		
HB* ~ AHI* + Age* + Asthma		
(*R* ^2^ = 0.40)		
AHI	0.64 (0.56–0.72)	< 0.001
Age	0.09 (0.01–0.16)	0.024
Asthma	−0.08 (−0.26;0.11)	0.400
Model 2		
HB* ~ AHI* + Obesity + Age* + Asthma		
(*R* ^2^ = 0.41)		
AHI	0.63 (0.54–0.71)	< 0.001
Obesity	0.23 (0.06–0.41)	0.008
Age	0.04 (−0.04; 0.12)	0.342
Asthma	−0.09 (−0.27; 0.10)	0.357
Model 3		
HB* ~ AHI* + Obesity + Tonsillar Hypertrophy + Age* + Asthma		
(*R* ^2^ = 0.42)		
AHI	0.60 (0.49–0.67)	< 0.001
Obesity	0.27 (0.09–0.44)	0.003
Tonsillar hypertrophy	0.33 (0.12–0.54)	0.002
Age	0.07 (−0.01; 0.15)	0.107
Asthma	−0.04 (−0.23; 0.14)	0.648

*Note*: *These variables were Box‐Cox transformed. The *λ* values (see Methods) that facilitated the normalisation of distributions were as follows: −0.5 for HB and AHI and 0.5 for age.

Abbreviations: AHI, apnea–hypopnea index; HB, hypoxic burden.

The prevalence of excessive daytime sleepiness, defined as a mESS score > 10 in the entire cohort, was 25% (95% CI: 22–29, *N* = 507). The mESS score was not correlated to the HB (rho_s_ = 0.05, *p* = 0.383), AHI (rho_s_ = −0.005, *p* = 0.919), or PLMI (rho_s_ = 0.02, *p* = 0.696). In univariate analysis, female sex was associated with increased mESS scores (8 [4; 12] vs. 6 [3; 10], *p* = 0.004), while asthma (*p* = 0.082), obesity (*p* = 0.128), and African ancestry (*p* = 0.261) were not associated with increased mESS. Among children with primary snoring, the prevalence of increased mESS was similar (26%, [95% CI: 21–32, *N* = 287]), and HB was associated with mESS (rho_s_ = 0.13, *p* = 0.048), while the AHI was not (rho_s_ = 0.10, *p* = 0.106). Fully adjusted multivariate analysis using a linear mixed‐effect model revealed that the HB in primary snoring children was associated with mESS; however, this was not the case when the analysis extended over the entire cohort of children with exploitable oximetry (Table 3). When replacing the HB with the AHI in the fully adjusted model, the AHI was not associated with mESS in children with primary snoring or the entire cohort of children with exploitable oximetry (Table S3).

**TABLE 3:** Exploratory analysis of the modified Epworth Sleepiness Scale (mESS) over children with primary snoring and over the entire cohort of children with exploitable oximetry.

Explicative variable*	mESS ~ variables + (1|subject) *N* = 225 (primary snoring)	mESS ~ variables + (1|subject) *N* = 380 (entire cohort)
Age, *y*	0.16 (0.03–0.29)	0.14 (0.03–0.25)
	*t*‐value = 2.4	*t*‐value = 2.6
	*p* = 0.018	*p* = 0.011
Sex (F vs. M)	0.41 (0.15–0.67)	0.23 (0.03–0.43)
	*t*‐value = 3.1	*t*‐value = 2.2
	*p* = 0.002	*p* = 0.028
Obese (yes vs. no)	*p* = 0.281	*p* = 0.326
Ethnicity—African‐Caribbean versus Caucasian	*p* = 0.443	*p* = 0.943
Asthma status, yes versus no	0.41 (0.12; 0.71)	*p* = 0.176
	*t*‐value = 2.8	
	*p* = 0.006	
Periodic limb movement index, h^−1^	*p* = 0.689	*p* = 0.809
Hypoxic burden, %min/h	0.16 (0.03; 0.29)	*p* = 0.720
	*t*‐value = 2.5	
	*p* = 0.013	
Marginal *R* ^2^	0.12	0.05
Conditional *R* ^2^	0.91	0.84

*Note*: *All continuous variables were zero centered and divided by the standard deviation over the whole cohort.

We evaluated the risk of having a mESS > 10 using a generalised mixed‐effect logistic regression analysis in the fully adjusted model in children with primary snoring and found an odds ratio for HB of 1.39 (95% CI, 1.01–1.89), while it was 0.94 (95% CI, 0.68–1.29) in the entire cohort of children with all levels of severity of SDB.

## 4 Discussion

In this study, we measured the HB in children and adolescents who snored, finding that HB was significantly associated with the severity of SDB. The primary contributors to HB were the AHI, obesity, and enlarged tonsils. The effects of obesity and tonsillar hypertrophy on the HB were reflected in the deeper desaturations observed in these children, independent of the AHI. Interestingly, some children with primary snoring exhibited significant HB, which was associated with sleepiness, likely due to desaturations associated with the deeper desaturations after respiratory events. It is also possible that some of these children experience desaturations associated with overall reduced respiratory flow that is not captured by the current definition of hypopnea or with short respiratory events, not responding to the apnea‐hypopnea definition in childhood (Sanchez‐Armengol et al. 1996).

HB was related to obesity and tonsillar hypertrophy independently of the AHI (Figures 1, 2, 3), with obesity and tonsillar hypertrophy associated with deeper desaturations. The deepness of an arterial desaturation after an apnea depends on the slope of the saturation versus time curve (${\dot{S}}_{a_{O_{2}}}$) and on the length of the apnea. As shown by (Sands et al. 2009), based on theoretical grounds, the three main determinants of ${\dot{S}}_{a_{O_{2}}}$ are the alveolar pressure of oxygen (PO_2_), the lung volume (approximated by the functional residual capacity), and the oxygen consumption. The first two factors are negatively associated with the slope (Bokov, Dudoignon, Matrot, and Delclaux 2025), while the last is positively associated. Thus, in obese children, deeper desaturations are expected as the slope of desaturation versus time curve is increased by at least two mechanisms: first, the resting alveolar PO_2_ is decreased in obese patients when compared to lean counterparts (Peters and Dixon 2018), and second, the functional residual capacity is also decreased by obesity (Peters and Dixon 2018). The effect of obesity on ${\dot{S}}_{a_{O_{2}}}$ is potentiated by the AHI, because in more severe OSAS, the resting arterial PO_2_ is further decreased (Sands et al. 2010). Furthermore, the BMI is positively associated with oxygen desaturation depth independent of age, sex, sleeping position, baseline SpO_2_, and event duration in adults (Peppard et al. 2009), further validating our results. Children with tonsillar hypertrophy tend to have more profound desaturations, likely due to increased plant gain (Armoni Domany et al. 2019)—although conflicting results were reported by our group (Bokov et al. 2022)—or longer apneas related to decreased loop gain (Bokov et al. 2022).

As expected, the HB correlated with the AHI. In our population, the correlation coefficient was approximately 0.6, which is lower than reported in adults (Azarbarzin et al. 2019). The HB is designed to capture the frequency of respiratory events and the depth and duration of the resulting desaturations. Thus, a higher AHI typically corresponds to a higher HB and vice versa. Previous studies in adults, including both OSA and non‐OSA populations, reported a correlation coefficient of 0.8 for the HB and AHI (Martinez‐Garcia et al. 2023). However, Azarbarzin et al. found that the correlations between HB and the conventional respiratory parameters, such as ODI, arousal index, wake time after sleep onset, and T90, were weaker in the group of patients with moderate to severe OSAS (Azarbarzin et al. 2019). This was not observed in our study and is probably explained by the less severe OSAS in children. Our distribution of the HB is quite similar to that reported by (Walter et al. 2025) in children with SDB, though our values are slightly higher. This difference is attributed to the calculation method used by Walter et al. which focused on respiratory event‐specific HB.

We observed a relationship between sleep hypoxic burden and daytime sleepiness only in children with primary snoring. One possible explanation is that hypoxic burden may exert a more noticeable impact on daytime sleepiness in these children because their sleep disruption is less severe and more directly linked to subtle variations in oxygen desaturation. In this group, even modest hypoxic episodes could disrupt wakefulness circuits and increase sleepiness, reflecting a sensitive physiological response. Conversely, in children with more severe sleep‐disordered breathing, such as OSAS, sleep disruption is typically profound and multifactorial, involving frequent arousals and fragmented sleep that may overshadow the specific influence of hypoxic burden. In such cases, overall sleep disruption—rather than hypoxic burden alone—may be the main contributor to daytime sleepiness, rendering the predictive value of hypoxic burden less evident. Additionally, it is possible that adaptive mechanisms in children with OSAS mitigate the impact of intermittent hypoxia on sleepiness (Sforza and Roche 2016).

A positive correlation between HB and the ESS score has been reported in adults (Esmaeili et al. 2023; Kainulainen et al. 2019). The HB odds ratio of 1.12 for excessive daytime sleepiness was reported in adults from a community‐based cohort. This could be interpreted as a 12% increase in the odds ratio for sleepiness for each SD increase of HB, estimated at 23.7% min/h (Esmaeili et al. 2023). The effects we report are much stronger since we evidenced a 39% increase in the odds ratio of excessive daytime sleepiness in children with primary snoring, with an increase of 1.5% min/h (1 SD) of HB. This result necessitates further investigation, especially when acknowledging that childhood SDB and daytime sleepiness predict children's reading ability (Joyce and Breadmore 2022) and that sleepiness has a strong negative impact on school performance (Dewald et al. 2010). Children with primary snoring exhibit reduced spindle activity when compared to healthy controls, which may be an indicator of sleep disruption and, therefore, could be involved in the development of disease‐related consequences (Brockmann et al. 2020). The prevalence of mESS > 10 that we found is similar to that reported by (Melendres et al. 2004) in children with SDB (28%), thus reinforcing the generalisability of our results.

Our study has potential clinical implications. In this work, we found that HB in children with primary snoring is related to cognitive deficits, thus possibly playing a role as the missing biomarker for intermittent hypoxia in children with mild SDB (Biggs et al. 2014). Hypoxic exposure via an increased HB in children with mild SDB could explain the increased cerebral blood flow velocity, obviating the group differences in cognitive function between children with mild OSAS and the non‐snoring controls (Hill et al. 2006).

Our study has some limitations. First, we used a non‐respiratory event‐specific method to calculate the HB, which differs from the more commonly used method described by Azarbarzin et al. (Azarbarzin et al. 2019). Second, when peripheral blood perfusion is compromised, finger pulse oximetry may not provide reliable pulsatile signals (photoplethysmography) that are essential to differentiate arterial blood and hence allow the accurate estimation of SpO_2_ (Kamat 2002). Another limitation comes from the use of the Nonin technology in most of the commercially available PSG devices. Indeed, (Blanchet et al. 2023) showed that differences of at least 4% between the estimated SaO_2_ (by the SpO_2_) and the value measured by arterial blood gas were present in 35% of the cases with the Nonin oximeter, which was the highest rate. This needs to be considered in further studies, especially in children where the rates and severity of arterial desaturations are less severe. Additionally, we used a version of the Epworth Sleepiness Scale that is less commonly employed than the ESS‐CHAD (Janssen et al. 2017), which might be viewed as a limitation. However, neither of the two scales has been validated for use in children or preschoolers. The French version of the ESS we utilised was validated in 384 adolescents and was demonstrated to be an effective tool for screening excessive daytime sleepiness in sleep disorders (Gustin et al. 2023). Another potential limitation was the use of parent reports of sleepiness and not an objective measure of sleepiness such as the Multiple Sleep Latency Test. However, no differences between self‐reports and parent reports were found for sleep duration and sleepiness in a recent meta‐analysis (Dewald et al. 2010).

## 5 Conclusion

In conclusion, our study examined the determinants and correlates of HB in children with SDB. We found that HB increased with the severity of SDB and identified obesity and tonsillar hypertrophy, in addition to the severity of SDB, as the primary contributors to elevated HB. HB was also related to excessive daytime sleepiness in children with primary snoring but not in children with OSAS, inviting us to reconsider theories of SDB‐related cognitive dysfunction in children.

## Author Contributions

**Plamen Bokov:** conceptualization, methodology, data curation, software, investigation, formal analysis, writing – original draft, visualisation, writing – review and editing. **Benjamin Dudoignon:** validation, investigation, writing – review and editing. **Christophe Delclaux:** methodology, formal analysis, writing – original draft, supervision, funding acquisition, project administration, resources, writing – review and editing.

## Disclosure

The authors have nothing to report.

## Ethics Statement

This study was approved by the local ethics committee (PHENOSAS: No. 2018‐416). The database of the collected data was registered with the French regulatory agency (CNIL). The participants and their parents were informed of the collection of the prospective data for research purposes, and they were given the option to opt out of the study in compliance with French law governing non‐interventional observational research.

## Conflicts of Interest

The authors declare no conflicts of interest.

## Supporting information


**Data S1:** jsr70211‐sup‐0001‐supinfo.docx.

## Data Availability

The data that support the findings of this study are available from the corresponding author upon request. The data are not publicly available because they contain information that may compromise the privacy of the research participants.

## References

[jsr70211-bib-0001] Armoni Domany, K. , Z. He , L. Nava‐Guerra , et al. 2019. “The Effect of Adenotonsillectomy on Ventilatory Control in Children With Obstructive Sleep Apnea.” Sleep 42: zsz045. 30805653 10.1093/sleep/zsz045

[jsr70211-bib-0002] Azarbarzin, A. , S. A. Sands , K. L. Stone , et al. 2019. “The Hypoxic Burden of Sleep Apnoea Predicts Cardiovascular Disease‐Related Mortality: The Osteoporotic Fractures in Men Study and the Sleep Heart Health Study.” European Heart Journal 40: 1149–1157. 30376054 10.1093/eurheartj/ehy624 PMC6451769

[jsr70211-bib-0003] Bacon, B. R. , A. B. Hassinger , G. Varavenkataraman , E. Gould , N. Sahlollbey , and M. M. Carr . 2024. “Comparison of Epworth Sleepiness Scale and OSA‐18 Scores With Polysomnography in Children.” Otolaryngology‐Head and Neck Surgery 171: 239–246. 38426572 10.1002/ohn.683

[jsr70211-bib-0004] Berry, R. B. , R. Brooks , C. Gamaldo , S. M. Harding , and American Academy of Sleep Medicine . 2017. The AASM Manual for the Scoring of Sleep and Associated Events: Rules, Terminology, and Technical Specifications. American Academy of Sleep Medicine.

[jsr70211-bib-0005] Biggs, S. N. , G. M. Nixon , and R. S. C. Horne . 2014. “The Conundrum of Primary Snoring in Children: What Are We Missing in Regards to Cognitive and Behavioural Morbidity?” Sleep Medicine Reviews 18: 463–475. 25060969 10.1016/j.smrv.2014.06.009

[jsr70211-bib-0006] Blanchet, M.‐A. , G. Mercier , A. Delobel , et al. 2023. “Accuracy of Multiple Pulse Oximeters in Stable Critically Ill Patients.” Respiratory Care 68: 565–574. 36596654 10.4187/respcare.10582 PMC10171338

[jsr70211-bib-0007] Bokov, P. , I. Boujemla , B. Matrot , K. Spruyt , J. Gallego , and C. Delclaux . 2022. “Oropharyngeal Obstruction and Respiratory System Compliance Are Linked to Ventilatory Control Parameters in Pediatric Obstructive Sleep Apnea Syndrome.” Scientific Reports 12: 17340. 36243786 10.1038/s41598-022-22236-7 PMC9569362

[jsr70211-bib-0008] Bokov, P. , B. Dudoignon , I. Boujemla , J. Dahan , K. Spruyt , and C. Delclaux . 2023. “Development and Validation of Moderate to Severe Obstructive Sleep Apnea Screening Test (ColTon) in a Pediatric Population.” Sleep Medicine 104: 11–17. 36870322 10.1016/j.sleep.2023.02.016

[jsr70211-bib-0009] Bokov, P. , B. Dudoignon , and C. Delclaux . 2025. “Hypoxic Burden as a Cause of Cardiovascular Morbidity in Childhood Obstructive Sleep Apnea.” Pediatric Research 104: 1–6. 10.1038/s41390-025-04153-3 PMC12920125 40410583

[jsr70211-bib-0010] Bokov, P. , B. Dudoignon , B. Matrot , and C. Delclaux . 2025. “Longer Respiratory Events in Childhood Obstructive Sleep Apnea Syndrome Constitute a Trait of Older Children With Excessive Daytime Sleepiness.” Scientific Reports 15: 4288. 39905224 10.1038/s41598-025-88876-7 PMC11794549

[jsr70211-bib-0011] Brockmann, P. E. , O. Bruni , L. Kheirandish‐Gozal , and D. Gozal . 2020. “Reduced Sleep Spindle Activity in Children With Primary Snoring.” Sleep Medicine 65: 142–146. 31869690 10.1016/j.sleep.2019.10.001

[jsr70211-bib-0012] Brodsky, L. 1989. “Modern Assessment of Tonsils and Adenoids.” Pediatric Clinics of North America 36: 1551–1569. 2685730 10.1016/s0031-3955(16)36806-7

[jsr70211-bib-0013] de Onis, M. , A. W. Onyango , E. Borghi , A. Siyam , C. Nishida , and J. Siekmann . 2007. “Development of a WHO Growth Reference for School‐Aged Children and Adolescents.” Bulletin of the World Health Organization 85: 660–667. 18026621 10.2471/BLT.07.043497 PMC2636412

[jsr70211-bib-0014] Dewald, J. F. , A. M. Meijer , F. J. Oort , G. A. Kerkhof , and S. M. Bögels . 2010. “The Influence of Sleep Quality, Sleep Duration and Sleepiness on School Performance in Children and Adolescents: A Meta‐Analytic Review.” Sleep Medicine Reviews 14: 179–189. 20093054 10.1016/j.smrv.2009.10.004

[jsr70211-bib-0015] Esmaeili, N. , G. Labarca , W.‐H. Hu , et al. 2023. “Hypoxic Burden Based on Automatically Identified Desaturations Is Associated With Adverse Health Outcomes.” Annals ATS 20: 1633–1641. 10.1513/AnnalsATS.202303-248OC PMC10632930 37531573

[jsr70211-bib-0016] Gozal, D. , M. Wang , and D. W. Pope Jr. 2001. “Objective Sleepiness Measures in Pediatric Obstructive Sleep Apnea.” Pediatrics 108: 693–697. 11533338 10.1542/peds.108.3.693

[jsr70211-bib-0017] Gustin, M.‐P. , B. Putois , A. Guyon , et al. 2023. “French Sleepiness Scale for Adolescents‐8 Items: A Discriminant and Diagnostic Validation.” L'Encéphale 49: 109–116. 10.1016/j.encep.2022.06.004 36253180

[jsr70211-bib-0018] Hill, C. M. , A. M. Hogan , N. Onugha , et al. 2006. “Increased Cerebral Blood Flow Velocity in Children With Mild Sleep‐Disordered Breathing: A Possible Association With Abnormal Neuropsychological Function.” Pediatrics 118: e1100–e1108. 17015501 10.1542/peds.2006-0092 PMC1995426

[jsr70211-bib-0019] Janssen, K. C. , S. Phillipson , J. O'Connor , and M. W. Johns . 2017. “Validation of the Epworth Sleepiness Scale for Children and Adolescents Using Rasch Analysis.” Sleep Medicine 33: 30–35. 28449902 10.1016/j.sleep.2017.01.014

[jsr70211-bib-0020] Joyce, A. , and H. L. Breadmore . 2022. “Sleep‐Disordered Breathing and Daytime Sleepiness Predict Children's Reading Ability.” British Journal of Educational Psychology 92: 576–593. 10.1111/bjep.12465 PMC9298025 34729766

[jsr70211-bib-0021] Kaditis, A. G. , M. L. A. Alvarez , A. Boudewyns , et al. 2016. “Obstructive Sleep Disordered Breathing in 2‐ to 18‐Year‐Old Children: Diagnosis and Management.” European Respiratory Journal 47: 69–94. 26541535 10.1183/13993003.00385-2015

[jsr70211-bib-0022] Kainulainen, S. , J. Töyräs , A. Oksenberg , et al. 2019. “Severity of Desaturations Reflects OSA‐Related Daytime Sleepiness Better Than AHI.” Journal of Clinical Sleep Medicine 15: 1135–1142. 31482835 10.5664/jcsm.7806 PMC6707054

[jsr70211-bib-0023] Kamat, V. 2002. “Pulse Oximetry.” Indian Journal of Anaesthesia 46: 261.

[jsr70211-bib-0024] Kim, J. S. , A. Azarbarzin , R. Wang , et al. 2020. “Association of Novel Measures of Sleep Disturbances With Blood Pressure: The Multi‐Ethnic Study of Atherosclerosis.” Thorax 75: 57–63. 31439722 10.1136/thoraxjnl-2019-213533 PMC8489982

[jsr70211-bib-0025] Lal, C. , T. E. Weaver , C. J. Bae , and K. P. Strohl . 2021. “Excessive Daytime Sleepiness in Obstructive Sleep Apnea. Mechanisms and Clinical Management.” Annals of the American Thoracic Society 18: 757–768. 33621163 10.1513/AnnalsATS.202006-696FR PMC8086534

[jsr70211-bib-0026] Martinez‐Garcia, M. A. , M. Sánchez‐de‐la‐Torre , D. P. White , and A. Azarbarzin . 2023. “Hypoxic Burden in Obstructive Sleep Apnea: Present and Future.” Archivos de Bronconeumología 59: 36–43. 36115739 10.1016/j.arbres.2022.08.005

[jsr70211-bib-0027] Melendres, M. C. S. , J. M. Lutz , E. D. Rubin , and C. L. Marcus . 2004. “Daytime Sleepiness and Hyperactivity in Children With Suspected Sleep‐Disordered Breathing.” Pediatrics 114: 768–775. 15342852 10.1542/peds.2004-0730

[jsr70211-bib-0028] Parekh, A. 2024. “Hypoxic Burden ‐ Definitions, Pathophysiological Concepts, Methods of Evaluation, and Clinical Relevance.” Current Opinion in Pulmonary Medicine 30: 600–606. 39229876 10.1097/MCP.0000000000001122 PMC11451971

[jsr70211-bib-0029] Parekh, A. , K. Kam , S. Wickramaratne , et al. 2023. “Ventilatory Burden as a Measure of Obstructive Sleep Apnea Severity Is Predictive of Cardiovascular and All‐Cause Mortality.” American Journal of Respiratory and Critical Care Medicine 208: 1216–1226. 37698405 10.1164/rccm.202301-0109OC PMC10868353

[jsr70211-bib-0030] Peppard, P. E. , N. R. Ward , and M. J. Morrell . 2009. “The Impact of Obesity on Oxygen Desaturation During Sleep‐Disordered Breathing.” American Journal of Respiratory and Critical Care Medicine 180: 788–793. 19644043 10.1164/rccm.200905-0773OC PMC2778152

[jsr70211-bib-0031] Peters, U. , and A. E. Dixon . 2018. “The Effect of Obesity on Lung Function.” Expert Review of Respiratory Medicine 12: 755–767. 30056777 10.1080/17476348.2018.1506331 PMC6311385

[jsr70211-bib-0032] Philbrook, L. E. , M. Shimizu , J. A. Buckhalt , and M. El‐Sheikh . 2018. “Sleepiness as a Pathway Linking Race and Socioeconomic Status With Academic and Cognitive Outcomes in Middle Childhood.” Sleep Health 4: 405–412. 30241654 10.1016/j.sleh.2018.07.008 PMC9204955

[jsr70211-bib-0033] Sanchez‐Armengol, A. , F. Capote‐Gil , S. Cano‐Gomez , R. Ayerbe‐Garcia , F. Delgado‐Moreno , and J. Castillo‐Gomez . 1996. “Polysomnographic Studies in Children With Adenotonsillar Hypertrophy and Suspected Obstructive Sleep Apnea.” Pediatric Pulmonology 22: 101–105. 8875583 10.1002/(SICI)1099-0496(199608)22:2<101::AID-PPUL4>3.0.CO;2-T

[jsr70211-bib-0034] Sands, S. A. , B. A. Edwards , V. J. Kelly , M. R. Davidson , M. H. Wilkinson , and P. J. Berger . 2009. “A Model Analysis of Arterial Oxygen Desaturation During Apnea in Preterm Infants.” PLoS Computational Biology 5: e1000588. 19997495 10.1371/journal.pcbi.1000588 PMC2778953

[jsr70211-bib-0035] Sands, S. A. , B. A. Edwards , V. J. Kelly , et al. 2010. “Mechanism Underlying Accelerated Arterial Oxygen Desaturation During Recurrent Apnea.” American Journal of Respiratory and Critical Care Medicine 182: 961–969. 20522790 10.1164/rccm.201003-0477OC

[jsr70211-bib-0036] Selvadurai, S. , G. Voutsas , S. L. Katz , H. Blinder , and I. Narang . 2022. “Evaluating Symptoms and Polysomnographic Findings Among Male and Female Children With Obesity With and Without Obstructive Sleep Apnea.” Sleep Medicine 100: 56–63. 36027663 10.1016/j.sleep.2022.07.013

[jsr70211-bib-0037] Sforza, E. , and F. Roche . 2016. “Chronic Intermittent Hypoxia and Obstructive Sleep Apnea: An Experimental and Clinical Approach.” Hypoxia 4: 99–108. 27800512 10.2147/HP.S103091 PMC5085272

[jsr70211-bib-0038] Snow, A. , E. Gozal , A. Malhotra , et al. 2002. “Severe Hypersomnolence After Pituitary/Hypothalamic Surgery in Adolescents: Clinical Characteristics and Potential Mechanisms.” Pediatrics 110: e74. 12456941 10.1542/peds.110.6.e74

[jsr70211-bib-0039] van Maanen, A. , A. H. Wijga , U. Gehring , et al. 2013. “Sleep in Children With Asthma: Results of the PIAMA Study.” European Respiratory Journal 41: 832–837. 22903967 10.1183/09031936.00019412

[jsr70211-bib-0040] Walter, L. M. , D. Bhatnagar , M. B. H. Ong , et al. 2025. “Sleep Apnea Specific Hypoxic Burden in Children With Down Syndrome and Typically Developing Children.” Journal of Sleep Research 34, no. 6: e70032. 40098587 10.1111/jsr.70032 PMC12592817

