# Supplementary material for: Hypoxic Burden in Children With Sleep‐Disordered Breathing: Determinants and Correlates
Source: J Sleep Res. 2025 Sep 20;35(2):e70211. doi: 10.1111/jsr.70211 (PMC13003301; doi:10.1111/jsr.70211)

**Supplementary Results**

**Table S1.** Demographic and sleep characteristics of children with available HB and those that were excluded from the analysis.

| Characteristic | Included patients  N=380 | Missing HB  N=132 | p-value |
| --- | --- | --- | --- |
| Age, years  Sex, male (%)  Ancestry, C/Af/A/M*  BMI Z-score   - Obese   Asthma, n (%)  Allergy, n (%)  AHI, h^-1^  OAHI, h^-1^  OAHI >5/h, n (%)  ODI_3%_, h^-1^  SpO_2_ min, %  T90, %  Arousal index, h^-1^ | 10.6 [7.5; 13.6]  209 (55%)  224/116/13/27  1.95 [0.60; 2.51]  185 (49%)  89 (23%)  118 (31%)  2.0 [1.0; 6.3]  1.1 [0.3; 5.3]  99 (26%)  1.7 [0.6; 4.3]  91 [89; 94]  0 [0; 0]  7.9 [5.4; 12.0] | 10.6 [7.8; 12.9]  86 (65%)  83/34/4/11  2.01 [0.81; 2.48]  66 (50%)  34 (26%)  38 (29%)  2.7 [1.0; 7.9]  2.0 [0.5; 7.2]  45 (34%)  3.6 [1.3; 9.1]  90 [87; 93]  0 [0; 0]  8.7 [5.8; 13.8] | 0.757  0.053  0.738  0.605  0.642  0.741  0.176  0.082  0.079  0.0002  0.004  0.105  0.105 |
| **ENT Examination** |  |  |  |
| Brodsky grade, 0/1/2/3/4 ^(missing data)^ | 99/125/78/57/19^(2)^ | 37/45/18/25/5^(2)^ | 0.415 |
| **Sleep Questionnaires** |  |  |  |
| mESS** | 7 [4; 11] | 7 [4; 10] | 0.884 |

Ethnicities are Caucasian/African-Caribbean/Asian/Mixed; BMI, body mass index; HB, hypoxic burden; AHI, apnea–hypopnea index; OAHI, obstructive apnea-hypopnea index, ODI_3%_, 3% oxygen desaturation index; SpO_2_, peripheral oxygen saturation; T90, total sleep time with oxygen saturation <90%; (N)REM, (non-) rapid eye movement; mESS, modified Epworth Sleepiness Scale; NT for not tested;

The median duration of a desaturation event was 25 s, with the 25^th^–75^th^ percentile ranging from 22 s to 31 s in the group of children with moderate to severe OSAS. The mean depth of desaturations was 2.9% [1.4; 4.6]. The estimated depth of desaturations may be less than 3% (the fixed minimal threshold) because when applying the formula 2*HB/(AHI*desaturation duration), the AHI includes some events that do not result in arterial desaturations.

**Table S2.** Linear mixed-effect models for desaturation depth and desaturation duration in children with moderate-to-severe OSAS.

| Model* | | Covariate Coefficients (standardized beta), 95% CI | | P-value |
| --- | --- | --- | --- | --- |
| Desaturation depth ~ AHI + Obesity + Tonsillar Hypertrophy + Age + (1\|Subject)   - AHI - Obesity - Tonsillar hypertrophy - Age   Marginal R^2^ = 0.12, Conditional R^2^ = 0.51 | 0.16 (-0.04; 0.36)  0.60 (0.15;1.06)  0.30 (-0.11;0.71)  0.00 (-0.23; 0.23) | | 0.130  0.013  0.153  >0.999 | |
| Desaturation duration ~ AHI + Obesity + Tonsillar Hypertrophy + Age + (1\|Subject)   - AHI - Obesity - Tonsillar Hypertrophy - Age   Marginal R^2^ = 0.04, Conditional R^2^ = 0.95 | -0.07 (-0.28; 0.14)  0.10 (-0.23; 0.43)  0.00 (-0.38; 0.38)  0.16 (-0.03; 0.35) | | 0.527  0.608  0.990  0.133 | |

* all continuous variables were zero centered and divided by the standard deviation over the whole cohort, in addition to that desaturation depth, desaturation duration, AHI and age were Box-Cox transformed. The λ values (see Methods) that facilitated the normalization of distributions were as follows: -0.5 for AHI, 0.5 for desaturation depth and age, and -1 for desaturation duration.

**Table S3.** Exploratory analysis of mESS over children with primary snoring and over the entire cohort of children with exploitable oximetry using AHI instead of HB.

| Explicative variable* | mESS ~ Variables + (1\|Subject)  N = 225 | mESS ~ Variables + (1\|Subject)  N = 380 |
| --- | --- | --- |
| Age, y | 0.17 (0.03-0.31)  t-value = 2.3  P = 0.020 | 0.15 (0.04-0.26)  t-value = 2.6  P = 0.010 |
| Sex (F vs M) | 0.38 (0.11-0.65)  t-value = 2.8  P = 0.006 | 0.23 (0.02-0.43)  t-value = 2.2  P = 0.032 |
| Obese (yes vs no) | P = 0.323 | P = 0.364 |
| Ethnicity  - African-Caribbean vs Caucasien | P = 0.572 | P = 0.993 |
| Asthma status, yes vs no | 0.40 (0.10-0.70)  t-value = 2.6  P = 0.01 | P = 0.182 |
| Periodic limb movement index, h^-1^ | P = 0.630 | P = 0.820 |
| AHI, event/hour | P = 0.875 | P = 0.680 |
| marginal R^2^  conditional R^2^ | 0.09  0.93 | 0.05  0.84 |

* all continuous variables were zero centered and divided by the standard deviation over the whole cohort

Sensitivity analysis of HB algorithm calculation to noisy SpO_2_ signals

To evaluate the sensitivity of the HB calculation algorithm, we artificially generated a 1-hour SpO_2_ signal sampled at 1 Hz and valued at 98%, containing 10 randomly distributed desaturations with durations ranging from 1 to 20 seconds, depending on the scenario being tested (see Table S4 and Figure 1S). All scenarios produced HB values consistent with the theoretically expected results, and the presence of artifacts did not affect the detection of desaturations.

Table 4S. Description of the artificial desaturation scenarios designed for artifact detection.

| Scenario | Length of desaturations | Number of desaturation sequences | SpO_2_ min | Theoretical HB, %min/h | Observed HB, %min/h |
| --- | --- | --- | --- | --- | --- |
| Desaturations | 10s | 10 | 80% | 15 | 15 (Figure 1S) |
| Ineligible desaturations | Random (1-20s) | 10 | 96% | 0 | 0 (Figure 2S) |
| Artifacts | Random (1-20s) | 10 | 0% | 0 | 0 (Figure 3S) |
| Artifacts | Random (1-20s) | 10 | Random (0-80%) | 0 | 0 (Figure 4S) |

Figure 1S. Hypoxic burden calculation according to the first scenario tested (see Table 4S). Desaturation detection (grayed area) in a 10s desaturations from 98 to 80%.


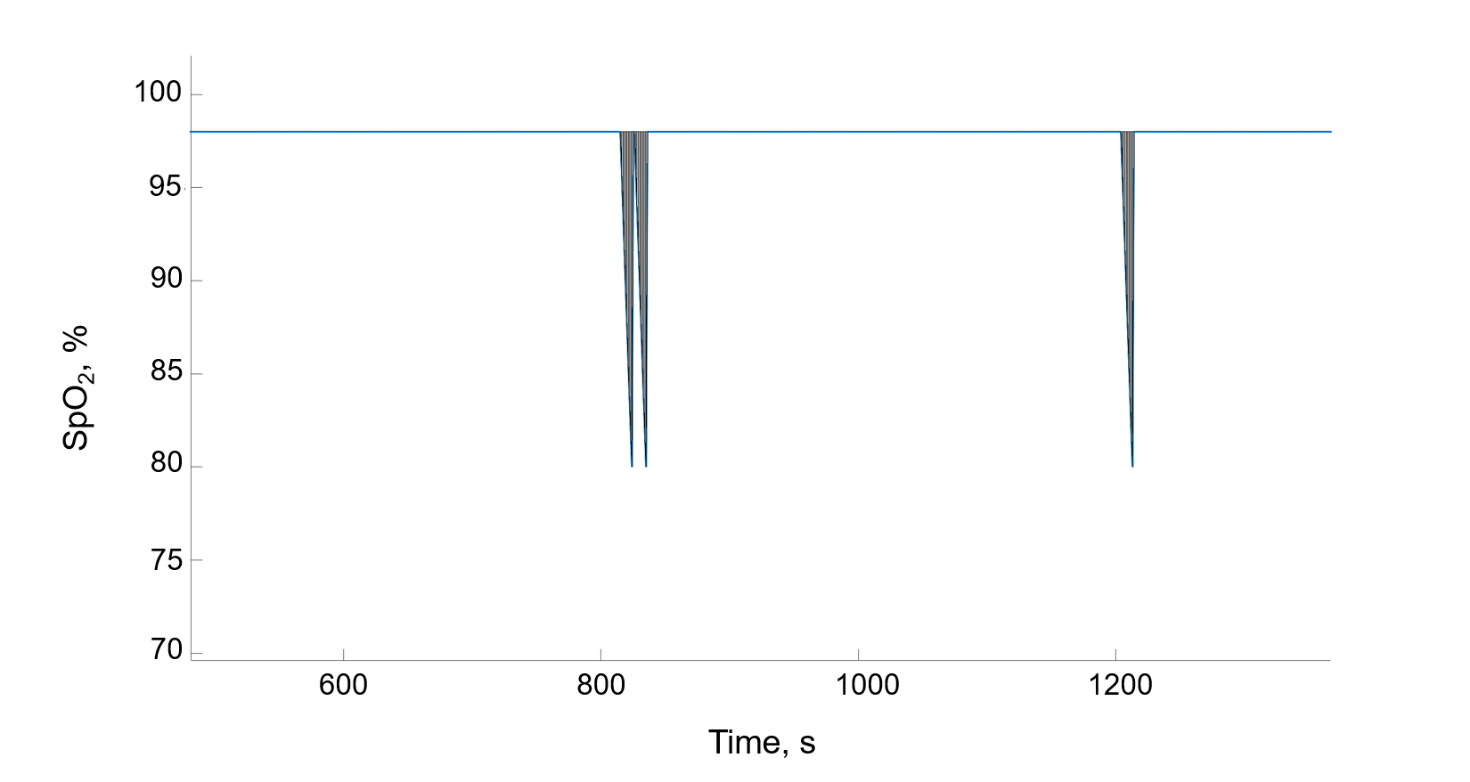


Figure 2S. Hypoxic burden calculation according to the second scenario tested (see Table 4S). Two points decreases of the saturation (from 98 to 96%) are not falsely detected.


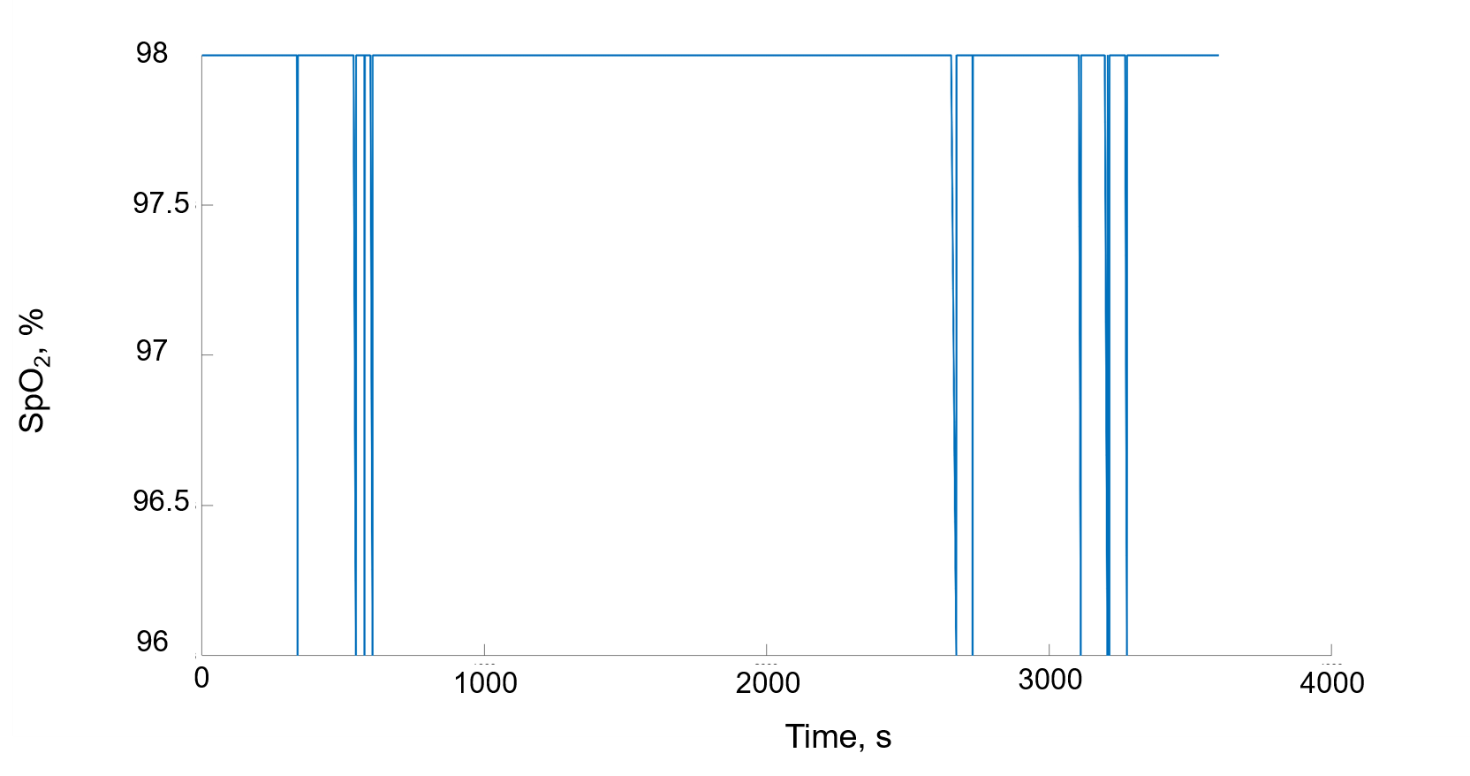


Figure 3S. Hypoxic burden calculation according to the third scenario tested (see Table 4S). Artefact desaturations to 0% are not falsely detected.


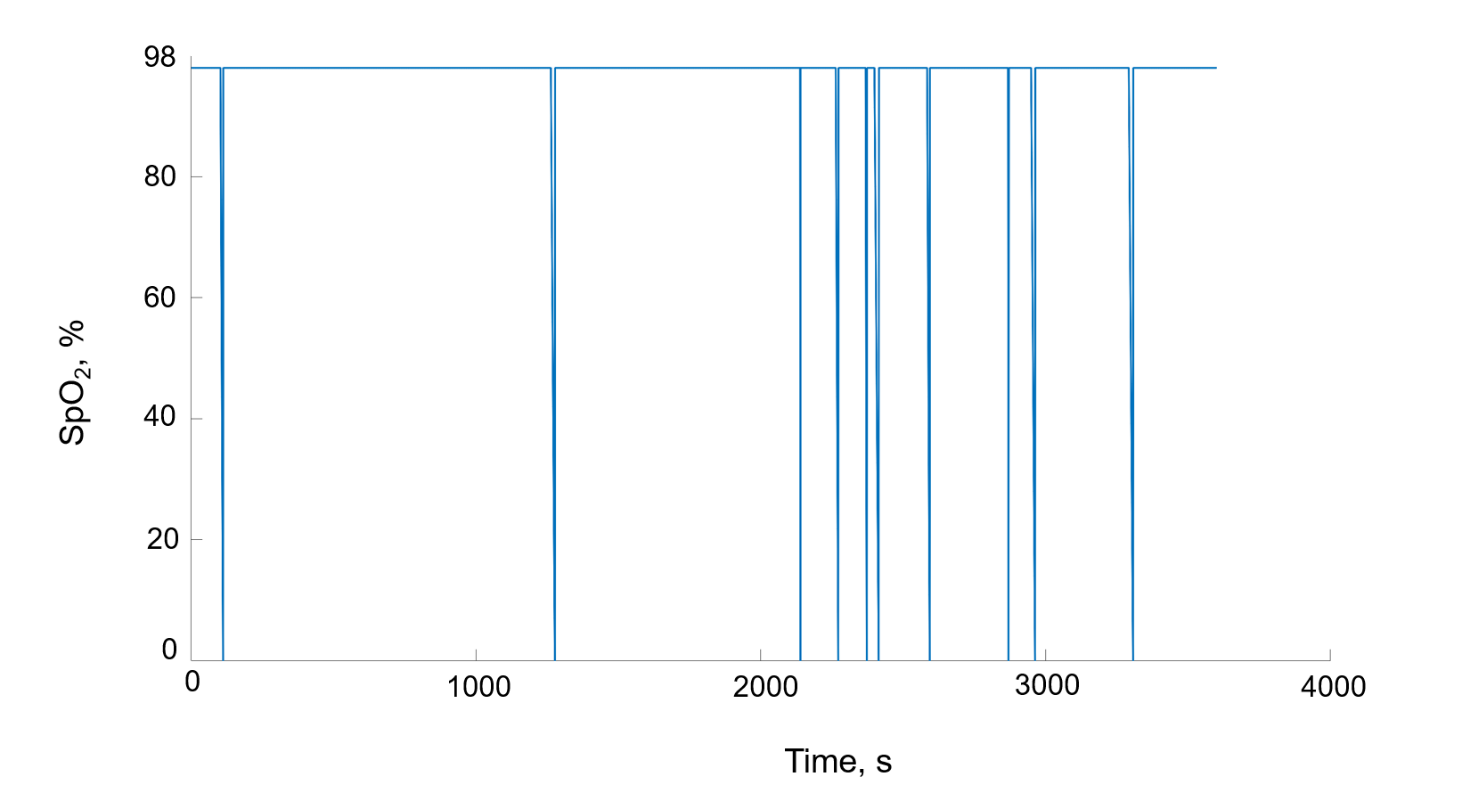


Figure 4S. Hypoxic burden calculation according to the third scenario tested (see Table 4S). Artefact random desaturations to SpO_2_ min from 80% to 0% are not falsely detected.


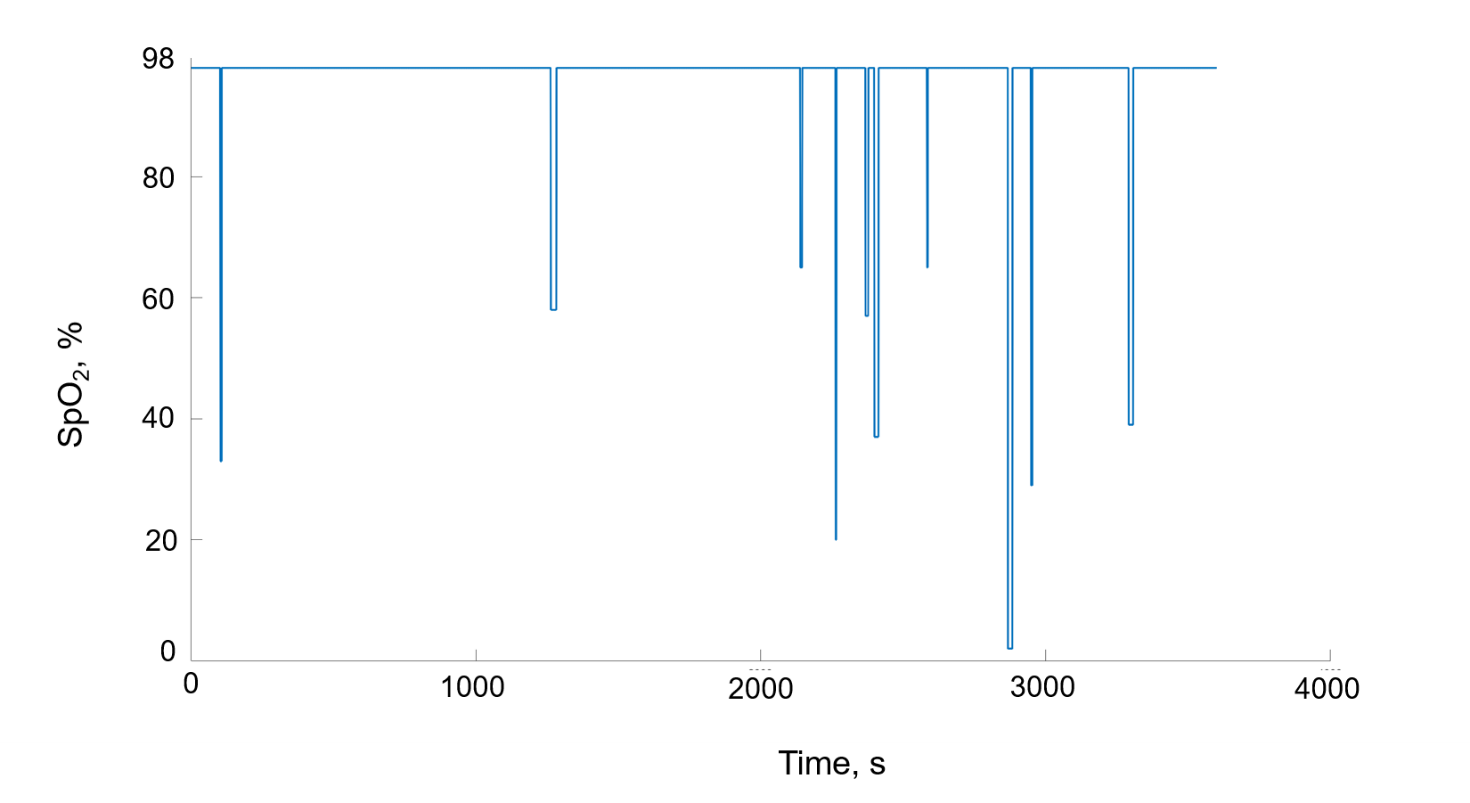

Supplement: Supplementary file 1 — Data S1: jsr70211‐sup‐0001‐supinfo.docx. [file JSR-35-e70211-s001.docx]
